# Supplementary material for: Gait analysis: An effective tool to mechanically monitor the bone regeneration of critical-sized defects in tissue engineering applications
Source: PLoS One. 2023 Dec 29;18(12):e0296510. doi: 10.1371/journal.pone.0296510 (PMC10756556; doi:10.1371/journal.pone.0296510)
Supplement: Attachment — Submitted filename: Response to reviewers.docx [file pone.0296510.s006.docx]

**Response to academic editor and reviewers**

**Academic editor**

**1. Please ensure that your manuscript meets PLOS ONE's style requirements, including those for file naming. The PLOS ONE style templates can be found at https://journals.plos.org/plosone/s/file?id=wjVg/PLOSOne_formatting_sample_main_body.pdf and https://journals.plos.org/plosone/s/file?id=ba62/PLOSOne_formatting_sample_title_authors_affiliations.pdf**

Thank you so much. The authors have rigorously reviewed the manuscript format according to the templates provided by the editor. Specifically, the tittle page, the headings and subheadings formatting, and the figure captions have been modified.

**2. As part of your revision, please complete and submit a copy of the Full ARRIVE 2.0 Guidelines checklist, a document that aims to improve experimental reporting and reproducibility of animal studies for purposes of post-publication data analysis and reproducibility: https://arriveguidelines.org/sites/arrive/files/documents/Author%20Checklist%20-%20Full.pdf Please include your completed checklist as a Supporting Information file. Note that if your paper is accepted for publication, this checklist will be published as part of your article.**

A copy of the Full ARRIVE 2.0 Guidelines checklist has been attached to the submission of the reviewed manuscript, and has been included in the Supporting Information: S5 File.

**3. Thank you for stating the following financial disclosure:**

**"This project was supported by the Ministerio de Ciencia e Innovación (Government of Spain) through the grant number PID2020-113790RB-I00 awarded to JMM and ERR. https://www.aei.gob.es/convocatorias/buscador-convocatorias/proyectos-idi-2020-modalidades-retos-investigacion-generacion "**

**Please state what role the funders took in the study. If the funders had no role, please state: "The funders had no role in study design, data collection and analysis, decision to publish, or preparation of the manuscript."**

**If this statement is not correct you must amend it as needed.**

**Please include this amended Role of Funder statement in your cover letter; we will change the online submission form on your behalf.**

The funders did not play a role in the experimental development of the study or in the process of publishing results. Therefore, the statement: "The funders had no role in study design, data collection and analysis, decision to publish, or preparation of the manuscript" was included in the cover letter of the reviewed manuscript.

**4. Thank you for stating the following in the Acknowledgments Section of your manuscript:**

**"This publication is part of the R+D project PID2020-113790RB-I00, financed by MCIN/AEI/10.13039/501100011033.'**

**We note that you have provided funding information that is not currently declared in your Funding Statement. However, funding information should not appear in the Acknowledgments section or other areas of your manuscript. We will only publish funding information present in the Funding Statement section of the online submission form.**

**Please remove any funding-related text from the manuscript and let us know how you would like to update your Funding Statement. Currently, your Funding Statement reads as follows:**

**"This project was supported by the Ministerio de Ciencia e Innovación (Government of Spain) through the grant number PID2020-113790RB-I00 awarded to JMM and ERR. https://www.aei.gob.es/convocatorias/buscador-convocatorias/proyectos-idi-2020-modalidades-retos-investigacion-generacion "**

**Please include your amended statements within your cover letter; we will change the online submission form on your behalf.**

The funding information stated in the Acknowledgments section of the manuscript corresponds to the same financing declared in the Funding Statement section. Therefore, the authors have simply removed the funding-related text in the new version of the manuscript. However, we would like to include a second project that contributed to some of the work materials. The new Funding statement should be:

"This project was supported by the Ministerio de Ciencia e Innovación (Government of Spain) through the grant number PID2020-113790RB-I00 awarded to JMM and ERR, https://www.aei.gob.es/convocatorias/buscador-convocatorias/proyectos-idi-2020-modalidades-retos-investigacion-generacion, and by the Junta de Andalucía (FEDER-UHU, Programa Operativo FEDER de Andalucía 2014-2020) through the grant number UHU-202058 awarded to JMM, https://www.agenciaandaluzadelaenergia.es/es/financiacion/incentivos-2017-2020/fondo-feder-2014-2020 ".

**5. In your Data Availability statement, you have not specified where the minimal data set underlying the results described in your manuscript can be found. PLOS defines a study's minimal data set as the underlying data used to reach the conclusions drawn in the manuscript and any additional data required to replicate the reported study findings in their entirety. All PLOS journals require that the minimal data set be made fully available. For more information about our data policy, please see http://journals.plos.org/plosone/s/data-availability.**

**"Upon re-submitting your revised manuscript, please upload your study's minimal underlying data set as either Supporting Information files or to a stable, public repository and include the relevant URLs, DOIs, or accession numbers within your revised cover letter. For a list of acceptable repositories, please see http://journals.plos.org/plosone/s/data-availability#loc-recommended-repositories. Any potentially identifying patient information must be fully anonymized.**

**Important: If there are ethical or legal restrictions to sharing your data publicly, please explain these restrictions in detail. Please see our guidelines for more information on what we consider unacceptable restrictions to publicly sharing data: http://journals.plos.org/plosone/s/data-availability#loc-unacceptable-data-access-restrictions. Note that it is not acceptable for the authors to be the sole named individuals responsible for ensuring data access.**

**We will update your Data Availability statement to reflect the information you provide in your cover letter.**

Following the journal’s requirements, the authors have added a Supplementary Information section in the reviewed manuscript to provide the minimal data set underlying our results, including:

S1 File. Outer geometry of a patient-specific scaffold. STL of the geometry of the scaffold designed from CT scans of an intermediate 13-mm ovine metatarsal bone fragment. The specific modifications made to the scaffold for stability and regenerative response are included.

S2 File. Control data of the stance phases. Ground reaction force data from the recorded stance phases in the non-operated control sheep of the study (n=3).

S3 File. Experimental data of the analyzed gait parameters. Complete set of all experimental data for all analyzed gait parameters (GRFpeak, GRFmean, t and Imp) in all animals in the study

S4 File. Asymmetries between hind- and forelimbs. Complete set of all data asymmetries in the analyzed gait parameters (GRFpeak, GRFmean, t and Imp) calculated between the hind- and forelimbs.

**6. Please review your reference list to ensure that it is complete and correct. If you have cited papers that have been retracted, please include the rationale for doing so in the manuscript text, or remove these references and replace them with relevant current references. Any changes to the reference list should be mentioned in the rebuttal letter that accompanies your revised manuscript. If you need to cite a retracted article, indicate the article's retracted status in the References list and also include a citation and full reference for the retraction notice.**

Thank you so much. A few references have been found to be not in the correct order according to the citation order in the manuscript, and they were corrected in the new version. However, all references have been checked so that they appear in the main text. New references were added to support the new information requested by the reviewers, but they have been marked in red color.

**Reviewer #1**

**This work evaluates the potential clinical use of gait analysis for the mechanical assessment of a tissue engineering regeneration as an alternative to the traditional and hardly conclusive manual or radiological follow-up. Nevertheless, there are areas that could benefit from refinement in order to enhance the overall clarity and precision of the manuscript.**

First of all, we would like to thank the reviewer for his/her suggestions and comments. From our point of view, they have substantially helped to improve the understanding of the purpose and the methodology of the study.

The responses to the comments of the reviewer are set out below. Reviewer’s comments are written in bold text font, and the authors’ responses are in normal font. Each response could include its own references placed at the end of the response. When referring to the manuscript, the authors have indicated the added lines in red color.

**Introduction:**

**Your introduction nicely emphasizes the role of tissue engineering in addressing critical-size bone defects. However, it would be great if you could clearly spell out how your study brings something new to the table in this field.**

**Line96-112”**

**The gait analysis is well-established. However, you could briefly highlight why gait analysis is a promising avenue for monitoring bone regeneration, especially in weight-bearing critical-sized defects.**

These first two comments from the reviewer will be answered at once. As mentioned by the reviewer, the first part of the introduction focuses on highlighting the advantages of tissue engineering for the repair of critical-size bone defects compared to grafting alternatives (allograft and autograft) or distraction approaches, including their versatility in reproducing the patient-specific segment geometry, the possibility of optimizing its internal microarchitecture to maximize regenerative response or the wide variety of biocompatible base materials available [1-5]. However, as pointed out in the manuscript, a “*continuous monitoring of the bone regeneration process is advisable, especially in the early weeks when bone infections or non-unions frequently appear*” (Introduction, lines 77-79). In the daily clinical routine, this monitoring is carried out using traditional techniques, such as plain film radiology, manual assessments, or qualitative mobility review, which often delays clinical decision making [6-9]. For example, radiology barely shows the formation of bone tissue until its mineralization, and manual methods tend to overestimate or underestimate the actual stiffness of the woven bone (Introduction, lines 77-91).

In the tissue engineering field, the opacity of its base material, its small pore sizes, and the generally high apparent stiffness of the structure could hinder performing a realistic analysis of the naïve tissue formation inside, especially in those critical first post-operative weeks. In the manuscript, this can also be observed in the x-rays of the Figure 3 (week 3). Thus, this research study establishes a methodology that opens the door to the use of gait analysis in clinics for more rigorous quantitative monitoring in patients with critical bone defects treated with tissue engineering. Compared with other mechanical characterization methods of bone regeneration, our approach is clinicians-friendly through low-cost devices whose collected data do not require complex post-processing tasks. Furthermore, the analyzed gait parameters (e.g., ground reaction force, contact time with the ground, impulse, or asymmetries) were demonstrated to evolve favorably over time and in line with the successful mineralization of the bone defect treated with bioceramic scaffolds radiologically. Our manuscript also delved into the potential correlation between the gait parameters in the operated and non-operated limbs throughout the callus consolidation phase. This interrelation could greatly simplify gait analysis in the clinical setting in contrast to the wide variety of parameters typically examined in biomechanical research studies. Moreover, according to the coefficient of determination shown in Figure 6 of the manuscript (R-square and p-value), we reported a significant relationship between parameters, especially in the treated limb. Lastly, our study brings to the table the idea of using gait parameters to compare scaffolds and bone regeneration processes in critical-sized defects from a new perspective. Beyond the typical comparisons in stiffness, bone mineral density, or volume of regenerated tissue [10-13], our study presents a methodology to compare processes in terms of the speed of patient mobility recovery, which is the ultimate goal of any regeneration. For example, the manuscript discusses and compares the evolution of the gait parameters with previous studies in bone transport in the same animal and bone model [10] (Discussion, lines 470-480).

As suggested by the reviewer, the authors include new lines in the manuscript to emphasize the novelty of the study in the field of tissue engineering: “*In the tissue engineering field, the opacity of its base material, its small pore sizes, and the generally high apparent stiffness of the structure could hinder performing a realistic analysis of the naïve tissue formation inside from radiological or manual assessments, especially in those critical first post-operative weeks*” (Introduction, lines 91-94); “*The proposed monitoring methodology is clinicians-friendly through low-cost devices whose collected data do not require complex post-processing tasks. This work also delved into the potential correlation between the different analyzed gait parameters in the operated and non-operated limbs throughout the TE mineralization phase as a means to simplify gait analysis in real clinical scenarios.*” (Introduction, lines 125-129).

References:

1. Lanza R, Langer R, Vacanti J P, Atala A. Principles of tissue engineering. Academic Press; 2020.
2. Bose S, Sarkar N, Banerjee D. Effects of PCL, PEG and PLGA polymers on curcumin release from calcium phosphate matrix for in vitro and in vivo bone regeneration. Mater Today Chem. 2018 Jun;8:110-120.
3. Blázquez-Carmona P, Sanz-Herrera JA, Martínez-Vázquez FJ, Domínguez J, Reina-Romo E. Structural optimization of 3D-printed patient-specific ceramic scaffolds for in vivo bone regeneration in load-bearing defects. J Mech Behav Biomed Mater. 2021 Sep;121:104613.
4. Sohn HS, Oh JK. Review of bone graft and bone substitutes with an emphasis on fracture surgeries. Biomater Res. 2019 Mar 14;23:9.
5. Eastaugh-Waring SJ, Joslin CC, Hardy JR, Cunningham JL. Quantification of fracture healing from radiographs using the maximum callus index. Clin Orthop Relat Res. 2009 Aug;467(8):1986-91.
6. Fisher JS, Kazam JJ, Fufa D, Bartolotta RJ. Radiologic evaluation of fracture healing. Skeletal Radiol. 2019 Mar;48(3):349-361.
7. Hammer RR, Hammerby S, Lindholm B. Accuracy of radiologic assessment of tibial shaft fracture union in humans. Clin Orthop Relat Res. 1985 Oct;(199):233-8.
8. Panjabi MM, Lindsey RW, Walter SD, White AA 3rd. The clinician's ability to evaluate the strength of healing fractures from plain radiographs. J Orthop Trauma. 1989;3(1):29-32.
9. Webb J, Herling G, Gardner T, Kenwright J, Simpson AH. Manual assessment of fracture stiffness. Injury. 1996 Jun;27(5):319-20.
10. Mora-Macías J, Reina-Romo E, López-Pliego M, Giráldez-Sánchez MA, Domínguez J. In Vivo Mechanical Characterization of the Distraction Callus During Bone Consolidation. Ann Biomed Eng. 2015 Nov;43(11):2663-74.
11. Horas K, Schnettler R, Maier G, Schneider G, Horas U. The role of soft-tissue traction forces in bone segment transport for callus distraction: A force measurement cadaver study on eight human femora using a novel intramedullary callus distraction system. Strategies Trauma Limb Reconstr. 2015 Apr;10(1):21-6.
12. Pobloth AM, Schell H, Petersen A, Beierlein K, Kleber C, Schmidt-Bleek K, Duda GN. Tubular open-porous β-tricalcium phosphate polycaprolactone scaffolds as guiding structure for segmental bone defect regeneration in a novel sheep model. J Tissue Eng Regen Med. 2018 Apr;12(4):897-911.
13. Blázquez-Carmona P, Mora-Macías J, Morgaz J, Fernández-Sarmiento JA, Domínguez J, Reina-Romo E. Mechanobiology of Bone Consolidation During Distraction Osteogenesis: Bone Lengthening Vs. Bone Transport. Ann Biomed Eng. 2021 Apr;49(4):1209-1221.

**Materials and Methods:**

**The reasoning behind selecting sheep as your animal model and a 15mm metatarsal defect as a critical-size bone defect needs more thorough explanation. Additionally, delving into the specifics of why you chose the particular bioceramic scaffold and external fixator would significantly enhance the manuscript. Providing more 3D-printing details on these decisions will** **enrich the comprehensibility of your research.**

In the last decades, mammalian animal models have become a benchmark for testing bone regeneration processes due to their ability to mimic human clinical cases. In the literature, the regeneration of long-bone defects has traditionally been investigated in several large species, such as sheep, dogs, or pigs [1]. Specifically, ovine models have several advantages over the other large-bone alternatives. They are comparatively docile and low-priced. A mature sheep also shears a comparable bone composition to humans [2] and a similar body weight, which enables a more straightforward extrapolation of the developed orthopedic methodologies [1]. Similarities in bone metabolic and remodeling rates were also reported by den Boer et al. [3]. Among all the ovine long bone models available, the metatarsus was chosen due to its thin layer of surrounding soft tissues, finding exclusively periosteal tissue on the lateral and medial sides. This anatomical advantage allowed a safer and more accessible tissue engineering surgery. The selection of the defect size was set for comparative reasons with other similar studies in the field. In ovine models, the critical defects chosen from other studies covering gait or bone callus mechanical analysis are around 5-20 mm [4-9]. Thus, choosing a 15-mm critical metatarsal defect allowed us to make the most rigorous comparison of the evolution of the analyzed gait parameters with other bone regeneration treatments in the same bone model and defect size [8].

Regarding the fixation, internal plates and splints are the classical stabilization methods surgically implemented in the critical-size weight-bearing bone defects treated [10-12]. They ensure a better bony alignment due to their stiffer assembly method. Nevertheless, external alternatives minimize body invasion through designs composed of rods or pins screwed into the treated bone and attached to external stabilizing rings. Hence, they reduce the soft tissue damage and blood loss during surgery. All this leads to an early recovery of the animals’ mobility and a lower impact on the analyzed gait parameters in the weeks immediately following the intervention. Lastly, external fixations are often used in bone regeneration studies to measure mechanical parameters through external instrumentation of the device [5-7, 9]. Following the same reason as the defect size, the discussion of the manuscript is more rigorous when compared to research studies that implement the same mechanical stabilization models.

Finally, scaffolds made of non-crystalline ceramics (e.g., hydroxyapatite) are proven to enhance naïve tissue growth by regulating osteoblast proliferation and differentiation [13]. Thus, they are gaining popularity compared to traditionally metallic alternatives that are more likely to block bone regeneration. Moreover, they are reported to have inherent biocompatibility, tissue conductivity, and reabsorbability through a “leave nothing behind” approach while new tissue grows [13].

Following the reviewer's suggestion, some lines were added to the manuscript to provide more details on the study's methodology and enrich the manuscript's comprehensibility: “*The selected mammalian model has numerous advantages. Sheep are docile, low-priced, and have a bone composition and body weight comparable to humans. These similarities allow for easier extrapolation of conclusions to real clinical cases [45-47]. The metatarsus was chosen as bone mode due to its thin layer of surrounding soft tissues, finding exclusively periosteal tissue on the lateral and medial sides. This anatomical advantage allowed a safer and more accessible tissue engineering surgery.*” (Materials and Methods, lines 144-149); “*For comparative reasons, this bone defect size was selected based on the size in other ovine bone regeneration experiments in the literature [4, 5, 39].*” (Materials and Methods, lines 156-157); “*This external solution minimizes body invasion compared to internal plates and splits. Hence, they reduce the soft tissue damage, leading to an early recovery of the animals’ mobility and a lower impact on the analyzed gait parameters in the weeks immediately following the intervention.*” (Materials and Methods, lines 159-162); “*This material was chosen since bioceramics are proven to enhance naïve tissue growth by regulating osteoblast proliferation and differentiation while the structure is reabsorbed [48].*” Materials and Methods, lines 186-188). New references were also added to assist this new information: references 45-48.

References:

1. Reichert JC, Saifzadeh S, Wullschleger ME, Epari DR, Schütz MA, Duda GN, Schell H, van Griensven M, Redl H, Hutmacher DW. The challenge of establishing preclinical models for segmental bone defect research. Biomaterials. 2009 Apr;30(12):2149-63. doi: 10.1016/j.biomaterials.2008.12.050.
2. Newman E, Turner AS, Wark JD. The potential of sheep for the study of osteopenia: current status and comparison with other animal models. Bone. 1995 Apr;16(4 Suppl):277S-284S. doi: 10.1016/8756-3282(95)00026-a.
3. den Boer FC, Patka P, Bakker FC, Wippermann BW, van Lingen A, Vink G QM, Boshuizen K, Haarman HJ Th M. New segmental long bone defect model in sheep: quantitative analysis of healing with dual energy x-ray absorptiometry. J Orthop Res. 1999 Sep; ;17(5):654-60. doi: 10.1002/jor.1100170506.
4. Brunner UH, Cordey J, Schweiberer L, Perren SM. Force required for bone segment transport in the treatment of large bone defects using medullary nail fixation. Clin Orthop Relat Res. 1994;301:147–155.
5. Forriol F, Goenaga I, Mora G, Vinolas J, Canadell J. Measurement of bone lengthening forces; an experimental model in the lamb. Cli Biomech. 1997; 12(1):17–21.
6. Meyers M, Schuelke J, Ignatius A, Claes L. Evolution of callus tissue behavior during stable distraction osteogenesis. J Mech Behav. Biomed. Mat. 2018; 85:12–19.
7. Mora-Macías J, Reina-Romo E, López-Pliego M, Giráldez-Sánchez MA, Domínguez J. In Vivo Mechanical Characterization of the Distraction Callus During Bone Consolidation. Ann Biomed Eng. 2015 Nov;43(11):2663-74.
8. Mora-Macías J, Reina-Romo E, Morgaz J, Domínguez J. In Vivo Gait Analysis During Bone Transport. Ann Biomed Eng. 2015 Sep;43(9):2090-100.
9. Blázquez-Carmona P, Mora-Macías J, Morgaz J, Fernández-Sarmiento JA, Domínguez J, Reina-Romo E. Mechanobiology of Bone Consolidation During Distraction Osteogenesis: Bone Lengthening Vs. Bone Transport. Ann Biomed Eng. 2021 Apr; 49(4):1209-1221.
10. Kon E, Salamanna F, Filardo G, Di Matteo B, Shabshin N, Shani J, et al. Bone regeneration in load-Bearing segmental defects, guided by biomorphic, hierarchically structured apatitic Scaffold. Front Bioeng Biotechnol. 2021; 27:734486.
11. Reichert JC, Epari DR, Wullschleger ME, Berner A, Saifzadeh S, Nöth U, at al. Bone tissue engineering. Reconstruction of critical sized segmental bone defects in the ovine tibia. Orthopade. 2012; 41(4):280–287.
12. Vidal L, Kampleitner C, Krissian S, Brennan MA, Hoffmann O, Raymond Y, et al. Regeneration of segmental defects in metatarsus of sheep with vascularized and customized 3d-printed calcium phosphate scaffolds. Sci Rep. 2020; 10:1–11.
13. Baino A, Novajra G, Vitale-Brovarone C. Bioceramics and Scaffolds: A Winning Combination for Tissue Engineering. Front Bioeng Biotechnol. 2015 Dec 17:3:202. doi: 10.3389/fbioe.2015.00202.

**Line152-154**

**Clarify the rationale behind the specific modifications made to the scaffold design for stability and regenerative response. Additionally, specify the reasons for selecting a 45 vol% hydroxyapatite slurry for the 3D-printing process and the significance of the resulting microarchitecture parameters.**

As commented by the reviewer, two modifications were performed on the original geometry of the scaffold, which was originally designed through multiplanar thresholding of CT-scanned intermediate 13-mm bone segments. A coupler cylinder (4 mm Ø; 2 mm length) was added over one end face. Its function is to improve the stabilization of the scaffold in the bone defect *in vivo*. As shown in Fig. R1, a scaffold non-attached to the hard tissue could move or rotate during the animal's daily activity. This is even more critical in our study since the defect is stabilized using an Ilizarov-type fixator. External fixations provide greater structural flexibility than internal alternatives and, allowing a more significant interfragmentary mobility of the remaining distal and proximal bone fragments. The potential movement of the structure *in vivo* could interfere with the proper growth of the naïve tissue. Thus, the coupler was introduced into the distal medullary cavity during surgery, thus minimizing its degrees of freedom. The second modification was digging a cylindrical hole (4 mm Ø; 10 mm length) at the other face end. Critical-sized bone defects tend not to heal spontaneously despite surgical stabilization [1]. The inclusion of an inorganic structure may not be sufficient on its own to ensure cell proliferation and subsequent tissue ingrowth. In the literature, authors tend to include bone morphogenetic proteins or grafted tissue to support and choreograph the tissue formation [2-4]. In our study, grafting tissue was cancellous bone autograft harvested from the lateral side of the contralateral humerus head (Materials and Methods, lines 227-231).

Regarding selecting a 45 vol% hydroxyapatite slurry, robocasting relies on the deposition through a nozzle of colloidal suspensions as a 3D-printing ink. This slurry should be capable of fully supporting its own weight during assembly to faithfully replicate the selected CAD design, in our case, the geometry of the scaffold. In the literature, other authors optimized the concentration of the colloidal suspensions to obtain the suitable viscoelastic properties of the ink for an effective deposition and assembly while ensuring the proper mechanical performance of the sintered structure *in vivo*. In calcium phosphate-based scaffolds, the powder contents of the inks are optimized in around 35-45 vol % of hydroxyapatite [5-7].

Finally, we cite a previous article where the microstructure of the scaffold is numerically optimized [8]: “*printing nozzle diameter, the pore size, or the layer overlap were numerically optimized to maximize cell diffusion and proliferation while ensuring the mechanical integrity of the structure under the ovine physiological loads. The final microarchitecture had a porosity of 59.3%, a 560.8 μm pore size, and a specific surface area of 5768.9 m^-1^*.” (Materials and Methods, lines 192-196). The optimized structural parameters are also discussed in the article mentioned above. Overall, these porosity, pore size, and specific surface area are in line with previous successful *in vivo* studies in tissue engineering [2-4].

**Figure R1**: Comparative diagram of the implantation of a scaffold without and with a medullary coupler in a critical bone defect of the ovine metatarsus.

An example of the final geometry of a patient-specific scaffold was added in the Supplementary information. Furthermore, some extra lines were added to the manuscript concerning this issue: “*This coupler would prevent the scaffold from rotating or moving during the animals’ daily activity, which could interfere with the proper growth of the naïve tissue.*” (Materials and Methods, lines 180-182); “*From previous works in the literature, the concentration of colloidal suspensions in the 3D-printing ink (45 vol%) was optimized to obtain the suitable viscoelastic properties for an effective deposition and assembly while ensuring the proper mechanical performance of the sintered structure in vivo [49-51]. An example of the final geometry of the patient-specific scaffold is provided in S1 File.”* (Materials and Methods, lines 188-192); “*Adding bone morphogenetic proteins or grafted tissue to the scaffold is an extended strategy used in tissue engineering experiments to avoid a lack of spontaneous healing in critical-size defects [21, 22].*” (Materials and Methods, lines 225-227). Regarding the optimization of the microstructure of the scaffold, the authors feel that it is redundant information when citing the specific research paper that addresses this issue. Therefore, no additional information was included in the manuscript.

References:

1. Nauth A, Schemitsch E, Norris B, Nollin Z, Watson JT. Critical-size bone defects: is there a consensus for diagnosis and treatment? J Orthop Trauma. 2018 Mar:32 Suppl 1:S7-S11. doi: 10.1097/BOT.0000000000001115.
2. Cipitria A, Reichert JC, Epari DR, Saifzadeh S, Berner A, Schell H, Mehta M, Schuetz MA, Duda GN, Hutmacher DW. Polycaprolactone scaffold and reduced rhBMP-7 dose for the regeneration of critical-sized defects in sheep tibiae. Biomaterials. 2013 Dec;34(38):9960-8. doi: 10.1016/j.biomaterials.2013.09.011. Epub 2013 Sep 24. PMID: 24075478.
3. Pobloth AM, Schell H, Petersen A, Beierlein K, Kleber C, Schmidt-Bleek K, Duda GN. Tubular open-porous β-tricalcium phosphate polycaprolactone scaffolds as guiding structure for segmental bone defect regeneration in a novel sheep model. J Tissue Eng Regen Med. 2018 Apr;12(4):897-911. doi: 10.1002/term.2446. Epub 2017 Jun 29. PMID: 28485078.
4. Vidal L, Kampleitner C, Krissian S, Brennan MÁ, Hoffmann O, Raymond Y, Maazouz Y, Ginebra M-P, Rosset P, Layrolle P. Regeneration of segmental defects in metatarsus of sheep with vascularized and customized 3D-printed calcium phosphate scaffolds. Sci Rep. 2020 Apr 27;10(1):7068. doi: 10.1038/s41598-020-63742-w.
5. Miranda P, Pajares A, Saiz E, Tomsia AP, Guiberteau F. Mechanical properties of calcium phosphate scaffolds fabricated by robocasting. J Biomed Mater Res A. 2008 Apr;85(1):218-27. doi: 10.1002/jbm.a.31587.
6. Miranda P, Saiz E, Gryn K, Tomsia AP. Sintering and robocasting of beta-tricalcium phosphate scaffolds for orthopaedic applications. Acta Biomater. 2006 Jul;2(4):457-66. doi: 10.1016/j.actbio.2006.02.004.
7. Indra A, Hamid I, Farenza J, Handra N, Subardi AA. Manufacturing hydroxyapatite scaffold from snapper scales with green phenolic granules as the space holder material. J Mech Behav Biomed Mater. 2022 Dec:136:105509. doi: 10.1016/j.jmbbm.2022.105509.
8. Blázquez-Carmona P, Sanz-Herrera JA, Martínez-Vázquez FJ, Domínguez J, Reina-Romo E. Structural optimization of 3D-printed patient-specific ceramic scaffolds for in vivo bone regeneration in load-bearing defects. J Mech Behav Biomed Mater. 2021 Sep;121:104613. doi: 10.1016/j.jmbbm.2021.104613. Epub 2021 Jun 8. PMID: 34126507.

**Line202**

**While the gait analysis section is detailed, consider briefly explaining the rationale behind choosing the specific gait parameters analyzed (GRF peak, GRF mean, tc, Imp) and their relevance to assessing the bone regeneration process.**

Although other biomechanical studies in the literature analyze a greater variety of kinematic gait parameters through complex electronic walkways in clinical cases applied to human fractures [1-3], our study bet for low-cost force plates as a simpler and more standardized alternative to real clinical scenarios. These devices only collect the evolution of the vertical load during the stance phase of the animal/patient, being the ground reaction force (*GRF* peak and mean), contact time with the ground (*t_c_*), the impulse (*Imp*), and the asymmetries the standard parameters to follow-up [2, 4-7]. As shown in Fig. R2, the healthy stance phase of an ovine hindlimb could actually be divided into four time-intervals based on the increasing and decreasing behavior of the ground reaction force curve (loading response, midstance, terminal stance, and preswing), as well as their two resulting ground reaction forces peaks. However, this differentiation does not apply to the analysis of any bone regeneration processes in critical-size defects since, as described in the manuscript, the pathological ground reaction force curve loses that M-shape to the greatest extent period of the bone consolidation phase.

**Figure R2**: Scheme of the four time-intervals in the stance phase of a healthy ovine hindlimb based on the increasing and decreasing behavior of the ground reaction force curve: loading response, midstance, terminal stance, and preswing.

In order not to overload information, the only thing added to the manuscript was the idea that the parameters are standard in this type of experiments: “All the above parameters are standard in gait analysis experiments with load platforms [24, 38, 39]” (Materials and Methods, lines 273-274).

References:

1. Warmerdam E, Orth M, Pohlemann T, Ganse B. Gait Analysis to Monitor Fracture Healing of the Lower Leg. Bioengineering (Basel). 2023 Feb 15;10(2):255. doi: 10.3390/bioengineering10020255. PMID: 36829749; PMCID: PMC9952799.
2. Larsen P, Laessoe U, Rasmussen S, Graven-Nielsen T, Berre Eriksen C, Elsoe R. Asymmetry in gait pattern following tibial shaft fractures - a prospective one-year follow-up study of 49 patients. Gait Posture. 2017 Jan;51:47-51. doi: 10.1016/j.gaitpost.2016.09.027. Epub 2016 Sep 28. PMID: 27701034.
3. Segal G, Elbaz A, Parsi A, Heller Z, Palmanovich E, Nyska M, Feldbrin Z, Kish B. Clinical outcomes following ankle fracture: a cross-sectional observational study. J Foot Ankle Res. 2014 Nov 28;7(1):50. doi: 10.1186/s13047-014-0050-9.
4. Mora-Macías J, Reina-Romo E, Morgaz J, Domínguez J. In Vivo Gait Analysis During Bone Transport. Ann Biomed Eng. 2015 Sep;43(9):2090-100.
5. Blázquez-Carmona P, Mora-Macías J, Morgaz J, Fernández-Sarmiento JA, Domínguez J, Reina-Romo E. Mechanobiology of Bone Consolidation During Distraction Osteogenesis: Bone Lengthening Vs. Bone Transport. Ann Biomed Eng. 2021 Apr; 49(4):1209-1221.
6. Fischer S, Anders A, Nolte I, Schilling N. Compensatory load redistribution in walking and trotting dogs with hind limb lameness. Vet J. 2013 Sep;197(3):746-52. doi: 10.1016/j.tvjl.2013.04.009. Epub 2013 May 15. PMID: 23683534.
7. Patterson KK, Nadkarni NK, Black SE, McIlroy WE. Gait symmetry and velocity differ in their relationship to age. Gait Posture. 2012 Apr;35(4):590-4. doi: 10.1016/j.gaitpost.2011.11.030. Epub 2012 Jan 31. PMID: 22300728; PMCID: PMC3914537.

**Line248**

**Elaborate on the frequency and specific aspects evaluated in the periodic X-rays. How did the X-ray observations correlate with the gait analysis findings, if at all?**

The authors thank the reviewer for this question. This research is only focused on evaluating the gait analysis as a low-cost methodology to assess the regeneration of bone defects in tissue engineering experiments. Therefore, the aim of this work is not to correlate the evolution in gait and x-ray parameters due to the limitation of this imaging technique as a quantitively method, as described in previous reviewer’s comments. Thus, the x-ray follow-up was performed monthly and, as stated in the manuscript, was only used for clinical monitoring of adequate regeneration, to ensure the non-appearance of non-unions, and to associate radiological changes to the evolution of gait parameters qualitatively. For instance, lines 306-308 of the discussion section associate the recovery of the ground reaction force after the initial lameness to “the gradual formation of a tissue bridge that consistently mineralized”. The “X-ray follow-up” section was rewritten in the manuscript to clarify it: “*In parallel with the gait analysis, the regeneration progress was verified through monthly x-rays of the operated metatarsal. These images were taken to clinically ensure the non-appearance of non-unions and to qualitatively associate changes detected in gait conditions with the level of ossification at the different regeneration stages.*” (Materials and Methods, lines 297-300).

**Results**

**Enhance the clarity of the results presentation by including specific numerical values for changes in gait parameters. This will assist readers in comprehending the magnitude of the observed alterations more effectively.**

The loading parameters analyzed in this manuscript (*GRF_peak_*, *GRF_mean_* and *Imp*) are directly dependent on the bodyweight of the animal, which varies considerably between study subjects (53.04 ± 6.34 kg). Therefore, it would not make sense to discuss the gait results in terms of loads in N without normalizing by the bodyweight. However, animal bodyweight data has been added to the manuscript (mean + std) so that future readers will be able to get an idea of the magnitudes: “*In general, the weight of the animals decreased from 57.90 ± 9.62 kg after surgery to 53.04 ± 6.34 kg before the sacrifice.*” (Results, lines 305-306). Additionally, some force data (N) has been included throughout the Results section (lines 339-364).

**Reviewer #2**

**This manuscript entitled "Gait analysis: an effective tool to mechanically monitor the bone regeneration of critical-sized defects in tissue engineering applications," evaluates the potential clinical use of gait analysis for the mechanical assessment of tissue engineering regeneration. The authors demonstrated that gait analysis is an effective, low-cost tool by conducting gait tests after metatarsal replacement in sheep. Here below are specific comments:**

We would like to thank the reviewer for his/her comments and suggestions on our manuscript. Some parts of the manuscript have been completed to consider his/her concerns.

The responses to the reviewer’s comments are set out below. The reviewer’s comments are written in bold text font and the authors’ responses are normal font. Each response could include its own references, which are placed at the end of the response. When referring to the manuscript, the authors have indicated the added lines by means of the section and line number. The authors have indicated these added words in the manuscript in red color.

**1. In the Introduction, the logic needs to be strengthened regarding the role of eliciting gait analysis in evaluating recovery after scaffolding treatment.**

A continuous monitoring of the bone regeneration process is needed in critical-size bone defects, especially in the early weeks when bone infections or non-unions frequently appear (Introduction, lines 77-79). In the daily clinical routine, this monitoring is carried out using traditional techniques, such as plain film radiology, manual assessments, or qualitative mobility review, which often delays clinical decision making [1-4]. For example, radiology barely shows the formation of bone tissue until its mineralization, and manual methods tend to overestimate or underestimate the actual stiffness of the woven bone (Introduction, lines 77-91).

In the tissue engineering field, the opacity of its base material, its small pore sizes, and the generally high apparent stiffness of the structure could hinder performing a realistic analysis of the naïve tissue formation inside, especially in those critical first post-operative weeks. In the manuscript, this can also be observed in the x-rays of the Figure 3 (week 3). Thus, this research study establishes a methodology that opens the door to the use of gait analysis in clinics for more rigorous quantitative monitoring in patients with critical bone defects treated with tissue engineering. Compared with other mechanical characterization methods of bone regeneration, our approach is clinicians-friendly through low-cost devices whose collected data do not require complex post-processing tasks. Furthermore, the analyzed gait parameters (e.g., ground reaction force, contact time with the ground, impulse, or asymmetries) were demonstrated to evolve favorably over time and in line with the successful mineralization of the bone defect treated with bioceramic scaffolds radiologically. Lastly, our study brings to the table the idea of using gait parameters to compare scaffolds and bone regeneration processes in critical-sized defects from a new perspective. Beyond the typical comparisons in stiffness, bone mineral density, or volume of regenerated tissue [5-8], our study presents a methodology to compare processes in terms of the speed of patient mobility recovery, which is the ultimate goal of any regeneration. For example, the manuscript discusses and compares the evolution of the gait parameters with previous studies in bone transport in the same animal and bone model [5] (Discussion, lines 470-480).

As suggested by the reviewer, the authors include new lines in the manuscript to emphasize the novelty of the study in the field of tissue engineering: “*In the tissue engineering field, the opacity of its base material, its small pore sizes, and the generally high apparent stiffness of the structure could hinder performing a realistic analysis of the naïve tissue formation inside from radiological or manual assessments, especially in those critical first post-operative weeks*” (Introduction, lines 91-94); “*The proposed monitoring methodology is clinicians-friendly through low-cost devices whose collected data do not require complex post-processing tasks. This work also delved into the potential correlation between the different analyzed gait parameters in the operated and non-operated limbs throughout the TE mineralization phase as a means to simplify gait analysis in real clinical scenarios.*” (Introduction, lines 125-129).

References:

1. Fisher JS, Kazam JJ, Fufa D, Bartolotta RJ. Radiologic evaluation of fracture healing. Skeletal Radiol. 2019 Mar;48(3):349-361.
2. Hammer RR, Hammerby S, Lindholm B. Accuracy of radiologic assessment of tibial shaft fracture union in humans. Clin Orthop Relat Res. 1985 Oct;(199):233-8.
3. Panjabi MM, Lindsey RW, Walter SD, White AA 3rd. The clinician's ability to evaluate the strength of healing fractures from plain radiographs. J Orthop Trauma. 1989;3(1):29-32.
4. Webb J, Herling G, Gardner T, Kenwright J, Simpson AH. Manual assessment of fracture stiffness. Injury. 1996 Jun;27(5):319-20.
5. Mora-Macías J, Reina-Romo E, López-Pliego M, Giráldez-Sánchez MA, Domínguez J. In Vivo Mechanical Characterization of the Distraction Callus During Bone Consolidation. Ann Biomed Eng. 2015 Nov;43(11):2663-74.
6. Horas K, Schnettler R, Maier G, Schneider G, Horas U. The role of soft-tissue traction forces in bone segment transport for callus distraction: A force measurement cadaver study on eight human femora using a novel intramedullary callus distraction system. Strategies Trauma Limb Reconstr. 2015 Apr;10(1):21-6.
7. Pobloth AM, Schell H, Petersen A, Beierlein K, Kleber C, Schmidt-Bleek K, Duda GN. Tubular open-porous β-tricalcium phosphate polycaprolactone scaffolds as guiding structure for segmental bone defect regeneration in a novel sheep model. J Tissue Eng Regen Med. 2018 Apr;12(4):897-911.
8. Blázquez-Carmona P, Mora-Macías J, Morgaz J, Fernández-Sarmiento JA, Domínguez J, Reina-Romo E. Mechanobiology of Bone Consolidation During Distraction Osteogenesis: Bone Lengthening Vs. Bone Transport. Ann Biomed Eng. 2021 Apr;49(4):1209-1221

**2. Lines 96-98: "As another option, gait analysis has been used to follow up on kinematics...", the reviewer agrees with this as gait analysis has become increasingly important in a variety of clinical assessments. perhaps the authors can refer to below research literature to further verify the opinion: (https://doi.org/10.1016/j.gaitpost.2023.10.019; https://doi.org/10.1038/s41598-022-07054-1 ).**

We thank the reviewer for providing new interesting references that bring to the table the impact of gait analysis in other research areas. They focused on investigating methodologies to select the best subset of biomechanical parameters to identify clinical gait pattern differences under specific study conditions. In particular, the second study employed Deep Neural Network (DNN) models and Layer-wise Relevance Propagation (LRP) techniques to identify differences in running gait patterns between higher-mileage and low-mileage runners. The authors agree that these references may help future manuscript readers better understand the potential of gait analysis in various clinical and biomechanical fields. Therefore, following the reviewer's suggestion, they were added to the new version of the manuscript: “*Among all the potentially collectible biomechanical parameters, some authors have developed algorithms to optimize the best set that identifies significant differences in gait patterns under the specific conditions of each study [41, 42].*” (Introduction, lines 108-110).

Xu D, Zhou H, Quan W, Jiang X, Liang M, Li S, Ugbolue UC, Baker JS, Gusztav F, Ma X, Chen L, Gu Y. A new method proposed for realizing human gait pattern recognition: inspirations for the application of sports and clinical gait analysis. Gait Posture. 2023 Oct 27:S0966-6362(23)01474-1. doi: 10.1016/j.gaitpost.2023.10.019.

Xu D, Quan W, Zhou H, Sun D, Baker JS, Gu Y. Explaining the differences of gait patterns between high and low-mileage runners with machine learning. Sci Rep. 2022 Feb 22;12(1):2981. doi: 10.1038/s41598-022-07054-1.

**3. Why were sheep chosen as subjects?**

Mammalian animal models are gaining popularity in this field due to their ability to mimic human clinical cases. In the literature, the regeneration of long-bone defects has traditionally been investigated in several large species, such as sheep, dogs, or pigs [1]. Ovine models have additional advantages over the other large-bone alternatives. They are comparatively low-priced and docile. A mature sheep also shears a comparable bone composition to humans [2] and a similar body weight, which enables a more straightforward extrapolation of the developed orthopedic methodologies [1]. Similarities in bone metabolic and remodeling rates were also reported by den Boer et al. [3]. Among all the ovine long bone models available, the metatarsus was chosen due to its thin layer of surrounding soft tissues, finding exclusively periosteal tissue on the lateral and medial sides. This anatomical advantage allowed a safer and more accessible tissue engineering surgery.

Following the reviewer's suggestion, some lines were added to the manuscript to provide more details on the study's methodology and enrich the manuscript's comprehensibility: “*The selected mammalian model has numerous advantages. Sheep are docile, low-priced, and have a bone composition and body weight comparable to humans [45-47]. These similarities allow for easier extrapolation of conclusions to real clinical cases. The metatarsus was chosen as bone mode due to its thin layer of surrounding soft tissues, finding exclusively periosteal tissue on the lateral and medial sides. This anatomical advantage allowed a safer and more accessible tissue engineering surgery.*” (Materials and Methods, lines 144-149). New references were also added to assist this new information: references 45-47.

References:

1. Reichert JC, Saifzadeh S, Wullschleger ME, Epari DR, Schütz MA, Duda GN, Schell H, van Griensven M, Redl H, Hutmacher DW. The challenge of establishing preclinical models for segmental bone defect research. Biomaterials. 2009 Apr;30(12):2149-63. doi: 10.1016/j.biomaterials.2008.12.050.
2. Newman E, Turner AS, Wark JD. The potential of sheep for the study of osteopenia: current status and comparison with other animal models. Bone. 1995 Apr;16(4 Suppl):277S-284S. doi: 10.1016/8756-3282(95)00026-a.
3. den Boer FC, Patka P, Bakker FC, Wippermann BW, van Lingen A, Vink G QM, Boshuizen K, Haarman HJ Th M. New segmental long bone defect model in sheep: quantitative analysis of healing with dual energy x-ray absorptiometry. J Orthop Res. 1999 Sep; ;17(5):654-60. doi: 10.1002/jor.1100170506.

**4. How is the gait phase determined? The gait of a sheep is different from that of a human, and this affects the trend of the ground reaction forces captured on the force plates.**

The reviewer is right. The ground reaction force curves in humans and ruminants are nonidentical since the distribution of bodyweight and physical loads radically differs between bipedal and quadrupedal subjects. However, the study does not aim to directly extrapolate the quantitative data to conclusions about bone regeneration in humans but rather to show the effectiveness of gait analysis in monitoring the regeneration of critical-size defects in tissue engineering experiments, including in human clinical cases.

Each gait phase for each of the animal's limbs was selected from the collected data from the time-point when an increase in ground reaction force was recorded to the time-point it returns to 0 N. Figure R3 shows an example of the raw loading records during stance phases of a right operated hindlimb and its ipsilateral one. During the experimental tests, a supervisor noted down the time-points in which each limb performs a complete stance phase on the force platform in order to associate load curves to its corresponding limb: the operated right hindlimb, the non-operated left hindlimb and their contralateral limbs. Moreover, as stated in the manuscript, “*collected stance phases performed above or below amble speed (2-4 km/hr) were visually discarded. Furthermore, data in which the animal interrupted its march at any point of the gangway were not included in the analysis either.”* (Materials and Methods, lines 256-259). Finally, an advantage of gait in ruminants is that, at an amble speed, the position on the ground where footsteps of forelimbs happen at the same location of the hindlimbs’ once. This also makes to identify gait phases in the collected raw data easier: a forelimb’s stance phase is followed by a hindlimb’s stance phase, as observed in Figure R3.

**Figure R3**: fragment of a ground reaction force (GRF) recording captured by the load platform in a gait test in sheep with critical-size bone defects treated with bioceramic scaffolds.

Some of this information was added to the manuscript: “*Each gait phase for every limb was determined by analyzing the collected data, starting from the time-point when an increase in ground reaction force was recorded until the time-point at which it returned to 0 N.”* (Materials and Methods, lines 254-256); “*During the experimental tests, a supervisor meticulously wrote down the specific time-points at which each limb executed a valid full stance phase on the load platform to further associate each GRF curve to their respective limb*.” (Materials and Methods, lines 259-261).

**5. Line 237: Please refer to the relevant studies.**

Thank you so much. References were added to assist in the formulation of the calculation of asymmetries between parameters: “*according to the formulation employed in previous works [39, 55]*” (Materials and Methods, lines 279-280). Moreover, a new reference was added to the manuscript (Reference 55):

Van Gelder LMA, Angelini L, Buckley EE, Mazzà C. A proposal for a linear calculation of gait asymmetry. Symmetry (MPDI). 2021 Aug;13(9):1560. Doi: 10.3390/sym13091560.

**6. Lines 244-245: For the description of linear regression models, please add the more detailed information (https://doi.org/10.1016/j.cmpb.2023.107761). For example, how to interpret the correlation coefficients, such as what is a high correlation and what is a low correlation.**

Additional information was included in the manuscript to detail the interpretation of the statistical coefficients used: “*The coefficient of determination* *R-squared is traditionally interpreted as the percentage of variance in one variable predicted or explained by the other [56]. In this field, authors tend to interpret R-squared over 0.49 and 0.81 as a strong and very strong correlation, respectively [5, 38, 39].* *The p-values for the coefficients indicate whether these previous relationships are or not statistically significant. Thus, a low p-value (< 0.01) would confirm that one gait parameter is significantly dependent of another.*” (Materials and Methods, lines 288-294). Moreover, a new reference was added to assist this new information, reference 56:

Schober P, Boer C, Schwarte LA. Correlation Coefficients: appropriate use and interpretation. Anesth Analg. 2018 May;126(5):1763-1768. doi: 10.1213/ANE.0000000000002864.

**7. The results section is best broken down into smaller points to expand on, which will make the overall logic and content clearer.**

We would like to thank the reviewer for this suggestion sincerely. Following the reviewer’s recommendation, the Results section is divided into subsections in the new version of the manuscript. The authors feel that it will improve the comprehension of the paper for future readers:

“*Gait parameters normalize over healing time*” (Results, line 303).

“*Gait asymmetries are attenuated during the regeneration*” (Results, lines 373).

“*Improvements in the gait of the different limbs are not independent*” (Results, line 389).

**8. The clarity of the pictures could be improved.**

The reviewer is right. When building the PDF of the manuscript through the Editorial Manager application, the figures (.png) lost resolution when converted to PDF format. The authors will try to improve this inconvenience during the submission of the reviewed manuscript.
